# A Teaching University for London

**Published:** 1894-04-07

**Authors:** 


### The Hospital

An Institutional Journal of

The Medical Sciences and Hospital Administration, with

Vol. XVI.—No. 393.] AN EXTRA NURSING SUPPLEMENT. [April 7, 1894.

### A Teaching University for London.

Academic subjects invite to academic methods of discussion. Unfortunately "academic" is too often the antithesis of "practical"; and when that is the case the psychological moment is frequently overlooked, and measures of profound and wide-reaching utility disappear for ever in the smoke of mere pedantic disputation. London never has had, has not now, but ought to have, a real and a great university. She has had an institution which has performed one function of a university, that of examining and labelling, exceedingly well. The presumption is that if facilities were granted to that institution it would perform other and much more important university functions equally well: that, indeed, the great critic might prove a great author, the competent examiner a liberal and successful educator.

The Royal Commission which was appointed in 1892, just about two years ago, to deal with the question of a teaching university for London, has issued its report, and educators whose aims are not only philosophical but immediately practical should make the report a starting-point for action which is designed to end in definite results. Mere discussion has lasted long enough; too long indeed for the patience of the public and the interests of the higher education. The Commissioners have made up their minds on certain fundamental points, and on those points the report speaks with no uncertain sound. Are we to have two universities for London or only one? "One," say the Commissioners. Is our one university to be a development of the existing university, or a creation of the *fin de siècle* brand new from the mint? It is to be a development of the now comparatively venerable institution which has attained to a fame that is world-wide, under the title of the "London University." These are fundamental points, and on these the Commissioners, as we have said, have absolutely made up their minds.

Now, obviously, these are points upon which all who have any concern in the matter should, like the Commissioners, make up their minds forthwith. For until they have so made up their minds, it is plain that they do not know the objective at which they want to arrive; and if a man does not know where he wants to go, it is exceedingly unlikely that he will ever get there. These matters, one would think, hardly require much discussion. A university is something like a great newspaper or a great business. If it has a reputation, more especially if it has a very great reputation, that reputation is simply priceless. No new concern can compare with it for a moment. If, then, the educators of London desire to have a university which shall at once be worthy of its name and of the metropolitan city of our vast empire, and if they have any of the instincts of practical statesmanship at all, they will resolve straightway to put their new venture to sea in the old vessel and under the old name and flag. For London there must be but one university; and that university must be a development of the existing and world-famous institution. On these points all persons of true academic tastes and of practical and statesmanlike instincts can have no opinion but one. If it be within the bounds of practical possibility at all, this is the objective to be aimed at.

But the making up of the mind as to an objective although it is one thing, and generally a fundamental thing, is not the same as arriving at the desired result. In the case under consideration, of one teaching university for London, the final result is a very great way off, and the practical difficulties are prodigious. But though that is so, the really statesmanlike mind will not for a moment swerve from its true objective, nor will it be at all dismayed by the practical difficulties. The objectors to the scheme, which has the approval of the Commissioners, of one teaching university, and that a development of the existing institution, will doubtless be as Babel both in the loudness and in the variety of their objections. But then it is to be remembered that any other scheme whatsoever, even though it should be evolved by the ghost of Socrates, propounded by that of Demosthenes, criticised by that of Whewell, and blessed by Huxley in the flesh, would have just as many objectors, and their objections would be just as diversified and irreconcileable. The truth is that when interests and individuals abound, and especially where those interests and individuals are of the academic type, there you have all the elements of a pyrotechnic demonstration, which may indeed have a beginning, but which knows neither middle nor end.

As a sample of the difficulties which stand in the way of the disestablishment of the existing London University as a mere examining board and its development into a great teaching institution with true university powers and functions may be mentioned the objection raised by certain medical teachers, that students, more especially medical students, ought not to be examined by their own professors and lecturers. This, we believe, is held to be one of the most serious of all the objections. But surely it is of no moment at all compared with the great object of securing a university which, as it would have the best material and the most unlimited facilities for study, would be able to command the ablest teachers in the world and the choicest pupils. If we grant that students, especially medical, should not be examined merely by their own teachers, it is open to us to point out that there can be no difficulty whatever in appointing supplementary examiners, and in appointing them in such numbers and of such competency that anything other than fair and competent examinations would be quite impossible. On this question it is sufficient to state that the University of Edinburgh, whose medical degrees have deservedly a world-wide reputation, has no difficulty whatever in securing fair and competent examinations of all the students that are taught by itself by means of combined professorial and extra professorial examinations; and, indeed, there is little doubt that the very fairest and most competent of all methods of examining students is this very method, which combines professorial with extra-professorial examination.

Another difficulty, and that of an important character, is the question of the examination and mint-stamping of students not educated either wholly or partly at the proposed London University itself. This difficulty has been emphasised in protestatory notes by several members of the Commission. But it ought not to be admitted as an insuperable difficulty. A university degree should, however, be a descriptive label of a university education, not merely of an examination, and the name of the university attached to a degree should imply that the student possessing such a degree possesses not merely the hall-mark, but the actual qualities and characteristics of that particular university and its education. A Doctor of Medicine of London should be a doctor of medicine who has the qualities and characteristics acquired by residence and education in London. Otherwise he is not really a doctor of medicine of London, but of Manchester, or Sheffield, or Leeds, as the case may be, and no hall-mark in the world can make him anything else. Surely the first condition of a university degree, which is a descriptive label of the person degreed, is that the label shall be truly and correctly descriptive of the person labelled.

These are samples of the practical difficulties which lie in the way of the supporters of the new scheme. How are they to be overcome? For that they shall be overcome is imperative if London and its higher education are not to be sacrificed to the incapacities of unpractical pedants. The Commissioners have shown us the way. "In view of the failure of previous attempts to settle this question," say they, "we are of opinion that in accordance with precedents . . . the changes should be effected, not by charter, but by legislative authority, and by the appointment of a commission with statutory powers." These are the words of practicality and of action. The first thing to be aimed at, we repeat, is the creation of a noble teaching university for the greatest teaching centre which civilisation can show; and the next is to give to the university the unique prestige which already adorns and has long adorned the examining body misnamed the "University of London." To these great objects all minor objects must be subordinated; and in order that they may be subordinated, and that the creation of the greatest of all universities, ancient or modern, may add one more to the splendours of a splendid age, we strenuously echo the practical recommendation of the Commissioners, that future action should be by way of effective legislation, and not by way of a mere permissive charter.

